# Shotgun Lipidomic Analysis for Differentiation of Niche Cold Pressed Oils

**DOI:** 10.3390/molecules27061848

**Published:** 2022-03-12

**Authors:** Hanna Nikolaichuk, Kacper Przykaza, Anna Kozub, Magdalena Montowska, Grażyna Wójcicka, Jolanta Tomaszewska-Gras, Emilia Fornal

**Affiliations:** 1 Department of Bioanalytics, Faculty of Biomedicine, Medical University of Lublin, Jaczewskiego 8b, 20-090 Lublin, Poland; hanna.nikolaichuk@umlub.pl (H.N.); annakozub@student.umlub.pl (A.K.); emilia.fornal@umlub.pl (E.F.); 2 Department of Meat Technology, Poznan University of Life Sciences, ul. Wojska Polskiego 31, 60-624 Poznan, Poland; magdalena.montowska@up.poznan.pl; 3 Department of Pathophysiology, Faculty of Medicine, Medical University of Lublin, Jaczewskiego 8b, 20-090 Lublin, Poland; grazyna.wojcicka@umlub.pl; 4 Department of Food Safety and Quality Management, Faculty of Food Science and Nutrition, Poznan University of Life Sciences, Wojska Polskiego 31/33, 60-624 Poznan, Poland; jolanta.tomaszewska-gras@up.poznan.pl

**Keywords:** camelina seed oil, flaxseed oil, hemp seed oil, authenticity, lipidomics, shotgun analysis

## Abstract

The fast-growing food industry is bringing significant number of new products to the market. To protect consumers’ health and rights, it is crucial that food control laboratories are able to ensure reliable quality testing, including product authentication and detection of adulterations. In our study, we applied a fast and eco-friendly method based on shotgun-lipidomic mass spectrometry for the authentication of niche edible oils. Comprehensive lipid profiles of camelina (CA), flax (FL) and hemp (HP) seed oils were obtained. With the aid of principal component analysis (PCA), it was possible to detect and distinguish each of them based on their lipid profiles. Lipidomic markers characteristic ofthe oils were also identified, which can be used as targets and expedite development of new multiplexed testing methods.

## 1. Introduction

Edible oils have been used since antiquity for health purposes; however, people did not realize what was responsible for this effect. Current analytical tools allow oils to be broken down into their individual components; thus, we can identify exact constituents affecting various medical conditions. Plant oils comprise mainly triacylglycerols and diacylglycerols (TAGs and DAGs), free fatty acids (FFA), phospholipids, glycolipids, carotenols, polyphenols, tocochromanols, phytosterols, carotenoids, and squalene, as well as antibacterial and anti-inflammatory ingredients [1]. They are characterized by their content of fat-soluble vitamins (A, D, E, K), n-9 monounsaturated fatty acids (MUFAs), and polyunsaturated fatty acids (PUFAs). These essential fatty acids must be obtained from the diet and are especially important for human health for the prevention of hypertension, coronary artery disease, diabetes, cancer, and many autoimmune inflammatory diseases (e.g., psoriasis, rheumatoid arthritis, Crohn disease, chronic obstructive pulmonary disease) [2]. Society is increasingly aware of the important health effects of vegetable oils. Moreover, the governments of highly developed countries are implementing circular economy models to reduce the amount of food waste by focusing on increasing consumption of plant seeds and pips [3], and the global production and consumption of plant oils are increasing. Unfortunately, along with the growing worldwide interest in vegetable oils, the risk of their adulteration also increases. This practice has been known for many years and, to date, has focused on extra-virgin olive oil due to its high price and consumption [4,5,6,7]. Many papers have focused on detecting the adulteration of edible fats and oils [5,8,9,10,11] using a wide range of analytical techniques, including infrared spectroscopy [12], nuclear magnetic resonance [13], differential scanning calorimetry [14], and chromatographic separation techniques (liquid or gas chromatography) coupled with various detectors (flame-ionization detectors, UV-Vis detectors, diodes, and mass detectors) [15,16,17,18]. The detection and determination of adulteration are generally based on measuring the amounts and ratio of TAGs and FAs coupled with chemometrics. However, profiling TAGs in edible oils and blends is challenging due to their very similar physicochemical properties and enormous variety, given the large number of FAs that constitute TAGs.

Triacylglycerols differ in the length and numbers of carbon chains, *cis/trans* configuration, the number and localization of unsaturated bonds, and the positions of substitutions on the glycerol backbone [19]. TAGs are often analyzed using high-performance liquid chromatography (HPLC) rather than gas chromatography (GC) because the derivatization step and high temperatures in GC analysis can degrade unsaturated TAGs. The main HPLC techniques used for separating TAGs are non-aqueous reverse-phase HPLC (NARP–HPLC) [20], silver-ion HPLC (Ag-HPLC) [21], and chiral liquid chromatography (chiral LC) [22]. Unfortunately, none of these techniques are perfect: NARP–HPLC has low selectivity towards compounds with identical ECN parameters, Ag-HPLC has low selectivity towards TAGs differing only in the length of the alkyl chain, and chiral LC requires partial hydrolysis of the acyl TAGs, precolumn derivatization, and ultrapure internal standards [23,24]. In contrast, DNA hybridization and polymerase chain reaction (PCR) are widely used in food analysis, especially in identifying counterfeit meat products [25]. However, although DNA markers are more specific than metabolic markers (they are unaffected by the cultivation region and conditions), highly processed matrices such as edible oils may undergo treatments that remove or damage DNA (e.g., degumming) during the oil refining process [26]. Alternative methods are thus required for the authentication of cooking and edible oils. Mass-detection analyzers are the gold standard for meticulous food authentication [27,28]. HPLC combined with mass detectors (HPLC–MS) provides a powerful analytical tool suitable for challenging and complicated biological matrices such as food. Mass-spectrometric methods provide a linear response and high sensitivity with both saturated and unsaturated TAGs [29]. Moreover, atmospheric-pressure chemical ionization (APCI) is frequently used for TAG analysis due to the large amount of information provided regarding the structure and position of fatty acids, its applicability to non-aqueous mobile phases, and most importantly, its ability to efficiently ionize non-polar particles [30]. Recently, the Gowda group [31] have used ultra-high-resolution liquid chromatography (UHPLC)–linear trap quadrupole (LTQ)–Orbitrap mass spectrometry analysis to identify FAs and profile FFAs in seafood. The advantages of the employed LC-MS method involve simple sample preparation and direct detection of FAs without any derivatization or chemical treatments. The method enabled the identification of FAs at trace levels. The main limitation of the method is that the evaluation of mass spectral fragmentation mechanism requires isotope-labeled experiments. Moreover, the quantification results are relative, not absolute, due to a lack of appropriate internal standards.

Han and Cheng (2005) developed a lipid analysis method based on direct infusion to the ion source sample delivery, namely “shotgun lipidomics” [32]. This feature allows maximum utilization of the unique chemical and physical properties of lipid classes, subclasses, and individual molecular species to facilitate the identification and quantification of lipids [32]. In addition, it ensures that the interactions between lipid species remain unchanged, which results in a constant amount of compound reaching the source, thereby leading to a constant ratio of ion peak intensities between classes of lipid species as well as constant suppression between compounds within a lipid class or between classes [33]. Another significant feature of shotgun lipidomics is the possibility of obtaining the entire mass spectrum of the sample with all molecular ions of a given class of lipids. This significantly facilitates the visualization and division of lipid species by mapping using fragmentation techniques (precursor ion scanning (PIS) and neutral loss scanning (NLS)), thereby assigning compounds to a specific lipid group or subgroup [34]. Shotgun lipidomics also enables the quantification of groups of compounds or individual chemical compounds present in the analyte by comparing the peak intensities with an internal standard [35]. Nowadays, shotgun lipidomics is successfully applied to determine lipids in various biological systems, including sentinel, mammalian, fish, plant species, blood plasma, tissues, food samples and yeast cells, for analytical, medical biological, food quality assessment purposes [36,37,38,39,40,41]. 

In recent years, niche oils, the oils pressed from non-traditional oil plants, have been gaining rapidly popularity due to the high content of various bioactive compounds, beneficial nutrition properties, pleasant taste and flavor. Clearly, there is a need to develop cost-effective, fast and reliable methods for distinguishing and authenticating niche edible oils, which are frequently adulterated, and at the same time, they have been beyond the focus of research till recently. Here, we present a mass spectrometry–shotgun analytical method of lipidomic profiling, originally designed for blood plasma analysis. In our experiment, we successfully applied this approach to characterize the lipid profiles (DAGs and TAGs) of three edible cold-pressed oils: camelina seed (CA), flaxseed (FL), and hemp seed (HP) oil. Several chemometric models for distinguishing oils based on the content of the molecular groups DAG and TAG, as well as molecular individual compounds, were created and described, and their applicability for oil identifications and authenticity testing were discussed. Finally, we identified DAG and TAG lipid markers, which can be used as detection targets in the process of the development of new methods for the identification and differentiation of these niche oils.

## 2. Materials and Methods

### 2.1. Samples

Brown flaxseeds of three different varieties were purchased from the Polish Institute of Natural Fibers and Medicinal Plants (Poznań, Poland), HodowlaRoślin STRZELCE Sp. z o.o. (Strzelce, Poland), Semco Sp. z o.o. manufactory (Śmiłowo, Poland), and Vitacorn Sp. z o.o. manufactory (Poznań, Poland). Camelina seeds of three different summer varieties were obtained from Semco Sp. z o.o. manufactory (Śmiłowo, Poland) and Poznań University of Life Sciences (Poznań, Poland). Hemp seeds were purchased from the Polish Institute of Natural Fibers and Medicinal Plants (Poznań, Poland). Seeds were pressed for oil at the Semco Sp. z o.o. manufactory (Śmiłowo, Poland)at a temperature under 50 °C. The pressed oils were left under nitrogen for 24 h for decantation and kept in brown glass bottles at −80 °C until analysis. In total, 5 camelina oils, 5 flaxseed oils and 5 hempseed oils were investigated.

### 2.2. Lipids Analysis

Lipids were determined by shotgun lipidomics analysis at Lipotype GmbH (Dresden, Germany). The applied assay allows the quantitation of lipids from twenty four classes: TAG, DAG, ceramides (CE), hexosylceramides (HexCer), phosphatidates (PA), phosphatidylcholines (PC), ether-linked PC (PC O-), phosphatidylethanolamines (PE), ether-linked PE (PE O-), phosphatidylglycerols (PG), phosphatidylinositols (PI), phosphatidylserines (PS), sphingomyelins (SM), cholesteryl esters (CE), cardiolipins (CA), lyso-phosphatidates (LPA), lyso-phosphatidylcholine (LPC), ether-linked LPC (LPC O-), lyso-phosphatidylethanolamine (LPE), ether-linked LPE (LPE O-), lyso-phosphatidylglycerols (LPG), lyso-phosphatidylinositols (LPI), and lyso-phosphatidylserines (LPS). Lipid class-specific internal standards arepresented in List S1 (Supplementary Materials).

Oils were diluted at 1:10,000 in two steps with a chloroform:methanol 1:1 (vol:vol) solution. Then, 100 μL of this dilution was used for the extraction. Lipids were extracted using one-step lipid extraction with methyl tert-butyl ether and methanol [36]. The samples were spiked with lipid class-specific internal standards prior to extraction. After drying and re-suspending in 7.5 mM ammonium acetate in chloroform/methanol/propanol (1:2:4 vol:vol:vol), the lipid extracts were subjected to mass spectrometric analysis. 

Mass spectra were acquired on a hybrid quadrupole/Orbitrap mass spectrometer (Thermo Fisher Scientific) equipped with an automated nano-flow electrospray ion source (Advion Biosciences) in both positive and negative ion modes, with a resolution of R*_m/z_*_=200_ = 280.000 for MS and R*_m/z_*_=200_ = 17.500 for MS/MS experiments. Five microliters of extracts were infused with gas pressure and voltage set to 1.25 psi and 0.95 kV, respectively. The delivery time was set to 4min and 55 s, with a contact closure delay of 20 s to avoid initial spray instability. Polarity switch from positive to negative mode was set at 135 s after contact closure. The samples were analyzed in both polarities in a single acquisition.

The MS acquisition method starts with positive ion mode by acquiring the *m/z* 402–412 in MS positive mode for 12 s. All individual scans in every segment are the average of 2 microscans. Automatic gain control (AGC) was set to 5 × 10^5^ and maximum ion injection time (IT) was set to 200 ms. Then, the scan of the *m/z* 550–1000 in MS positive mode with lock mass activated at a common background (*m/z* = 680.48022) followed for 18 s. AGC was set to 1 × 10^6^ and IT was set to 50 ms. This was followed by a MS/MS positive mode data-independent analysis triggered by an inclusion list for 105 s. The inclusion list contains all the masses from 500.5 to 999.75, with 1 Da intervals. AGC was set to 10^5^ and IT was set to 64 ms. The isolation width was set to 1Da, the first mass of MS/MS acquisition was 250 Da and normalized collision energy was set to 20%. Both MS and MS/MS positive mode data are combined to monitor SE, DAG, and TAG ions as ammonium adducts. After the polarity switch to negative ion mode, a lag of 15 s before acquisition was inserted to allow spray stabilization. Then, the scan for the *m/z* 400–650 in FTMS was executed for 15 s with lock mass activated at a common background (*m/z* = 529.46262) to monitor LPG, LPA, LPI, LPS, and LPE as deprotonated anions and LPC and LPC O– as acetate adducts. AGC was set to 10^6^, and IT was set to 50 ms. Then, the scan of the *m/z* 520–940 in FTMS followed for 15 s, with lock mass activated at a common background (*m/z* = 529.46262). AGC was set to 10^6^, and IT was set to 50 ms. Finally, the scan MS/MS in negative mode was acquired by data-independent analysis triggered by an inclusion list for 90 s. This inclusion list contains all the masses from 590.5 to 939.5, with 1 Da intervals. AGC was set to 10^5^, and IT was set to 64 ms. Isolation width was set to 1 Da, the first mass of MS/MS acquisition was 150 Da, and normalized collision energy was set to 35%. Both MS, and MS/MS data were combined in order to monitor PC, PC O–, HexCer, Cer and SM as acetate adducts and PS, PG, PA, PE, PE O– and PI as deprotonated anions. Lipid identification was performed on unprocessed raw mass spectra using LipotypeXplorer [42]. For the MS-only mode, lipid identification was based on the molecular masses of the intact molecules. The MS/MS mode included the collision-induced fragmentation of lipid molecules, and lipid identification was based on the parent and daughter ions. Lipids were filtered according to accurate mass, ion abundance threshold, noise, and background before normalization and further data processing. Lipids with an intensity 5-fold greater than noise in mass spectrum and 5-fold greater than the intensity of blank samples were subjected to identification. Lists of identified lipids and their intensities were stored in a database optimized for the particular structure inherent to lipidomic datasets. The intensity of lipid class-specific internal standards was used for lipid quantification. Without fragmentation, high-resolution MS allowed us to assign the sum composition of a lipid molecule to a peak in the spectrum. Fragmentation of the molecule revealed the fatty acid composition. Lipids were annotated at confidence level 2, putative identification [43]. The identified lipid molecules were quantified by normalization to a lipid class-specific internal standard. The internal standard signal-to-noise ratio was in a range of hundreds and more, indicating high quality of spectra. The technical reproducibility, as assessed by triplicates of one oil sample as reference samples included in the same analytical run, was very good, with the median coefficient of variation across all lipid classes being 9.1% (5.8% and 12.4% for TAG and DAG, respectively). The recovery of each internal standard was in the acceptance range of 80–120%.

Detailed information on the Lipotype shotgun lipidomics method, including data acquisition, data processing, and lipid quantification procedure is presented in the paper by Surma et al. [36]. 

The amounts of individual lipid molecules and lipid molecular groups are reported in µmol/mL.

### 2.3. Multivariate Data Analysis

Principal component analysis (PCA) and orthogonal partial least square discriminant analysis (OPLS-DA) were carried out using SIMCA software version 16.1 (Sartorius Stedim Data Analytics AB, Umea, Sweeden). PCA and OPLS-DA modeling were performed on following datasets: DAG molecular groups, TAG molecular groups, DAG subspecies, combined DAG and TAG molecular species, and fatty acid content in DAG and TAG; the average values and standard deviations for these data are reported in Tables S1–S3 (Supplementary Materials). Data preprocessing involved Pareto scaling and centering. The models were cross-validated (PCA, OPLS-DA) and validated by permutation testing (OPLS-DA). The quality of models was assessed based on model statistics R2X, R2Y, S2Y, Q2 and SEE from cross-validation;model performance was evaluated by considering the explained variation R^2^—goodness of fit for X- and Y-variables, respectively, the predictive variation Q^2^—goodness of prediction, the fit for predicted variables, the variance of Y-matrix (S^2^Y), i.e., the residual (not modelled) variance of Y and the standard error of estimate (S.E.E.)—a root-mean-square error of estimates.

### 2.4. Results

#### 2.4.1. Characteristics of DAG and TAG Profiles of Oils

The analysis of three cold-pressed edible oils CA, FL and HP using shotgun mass spectrometry provided their comprehensive quantitative DAG and TAG profiles (Tables S1 and S2, Supplementary Data), as well as the distribution of FAs in DAG and TAG (Table S3, Supplementary Data). DAGs were identified to subspecies level (individual molecules), TAG to species level (molecular groups), i.e., lipid species were annotated according to their molecular composition as name (DAG or TAG) sum of the carbon atoms in the hydrocarbon moiety: sum of the double bonds in the hydrocarbon moiety (e.g., TAG 50:1, DAG 36:0). Lipid subspecies annotation contains additional information on the exact identity of their acyl moieties (e.g., DAG 18:1/16:0). Thus, for DAGs, two sets of data were available: individual molecules (e.g., DAG 14:0/18:3) and molecular groups (e.g., DAG 32:3). In the case of TAGs, only molecular groups were obtained (e.g., TAG 50:1). Table 1 presents the number of molecular groups of DAG and TAG, and DAG individual molecules detected in camelina, flax and hemp seed oils.

**Figure 1:**
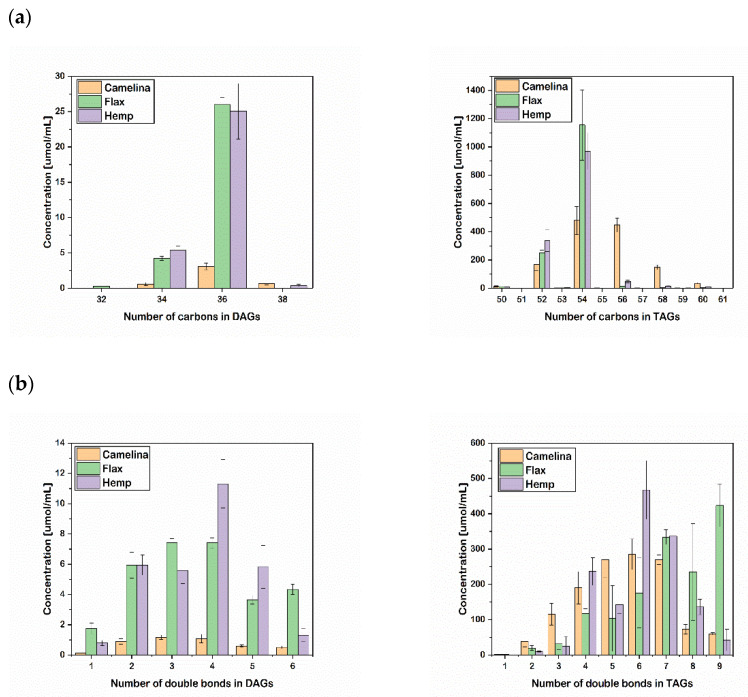
DAG and TAG composition based on the number of (**a**) double bonds and (**b**) carbon atoms.

Analyzed oils were characterized by their DAG and TAG profiles, making it possible to distinguish them on several levels. Figure 1 shows differences in the number of double bonds and the total number of carbons in the FA chains of DAGs and TAGs.

CA oil had a lower content of MUFAs and PUFAs in DAGs compared to FL and HP oils. The MUFA content of DAGs was estimated at 0.1 µmol/mL, and the PUFA content (number of double bonds 2–6) in DAGs was similar for each group of double bonds and reached a maximum (1.2 µmol/mL) for three double bonds in two fatty-acid chains. FL and HP oils were characterized by a much higher PUFA content in DAGs (number of double bonds 2–6), with the concentration being 3–7 times higher. HP oil had the highest DAG content with four double bonds (11.3 µmol/mL), and FL oil had the highest DAG content (4.3 µmol/mL), with six double bonds in the FAs (Figure 1). 

Content of MUFAs in TAGs was negligible compared to PUFAs in each of analyzed oil (CA, 2.2 μmol/mL; FL, 1.8 μmol/mL; HP, <0.1 μmol/mL). CA oil had the highest TAG content with group of two, three and five double bonds in three fatty-acid chains. The concentration of PUFAs in FL oil increased with the number of double bonds, reaching a maximum for nine double bonds in the FAs (423.6 μmol/mL), which is much higher compared to CA (60.3 μmol/mL) and HP oils (42.5 μmol/mL) (Figure 1a). DAGs with 34 and 36 carbons were present at higher concentrations in HP and FL oils than in CA oil, whereas CA oil had the highest content of DAGs containing 38 carbons (0.7 µmol/mL), and only FL oil contained DAGs with 32 carbons (0.3 µmol/mL) (Figure 1b). TAGs with 54 carbons were the highest in all three oils, and FL and HP had twice the content of CA oil. The FL and HP oils contained TAGs with 52 and 54 carbons, and only CA oil contained TAGs with 50 to 61 carbons. Simultaneously, CA oil revealed significantly higher concentrations of TAGs with 56, 58, and 60 carbons compared to FL and HP oils, showing that this oil is the richest source of TAG lipid species of the studied oils.

#### 2.4.2. Multivariate Data Analysis to Differentiate CA, FL, and HP Oils

The complexity of the lipidomic data does not allow direct comparison of DAG and TAG concentrations for the classification and differentiation of the analyzed oils. To assess the DAG and TAG profile discriminating potential, and to determine how deep characterization—to species or subspecies level, combining the two or using only one, i.e., DAG or TAG profile—is required for the successful classification and discrimination of the oils, first, the PCA models were build using lipid features from both levels (individual molecule datasets and molecular group datasets) as well as FA profile in DAG and TAG. Principal component analysis (PCA) is the most commonly applied multivariate projection method designed for extracting and displaying the systematic variation in a data matrix, which helps to identify the correlation structure in a datasets.

**Figure 2:**
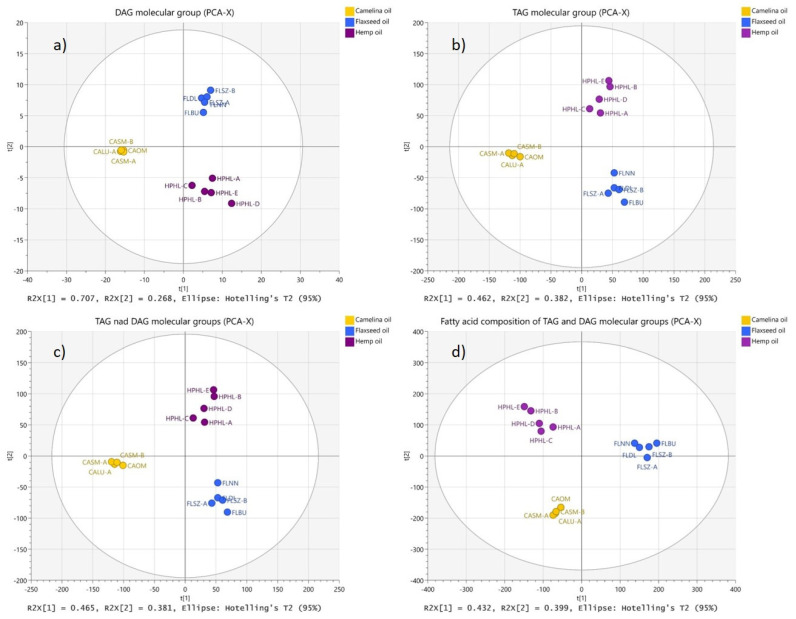
PCA score plots of the oil lipid composition data: (**a**) DAG molecular groups (principal component explained variance (PC %EV) = 97.5%; (**b**) TAG molecular groups (PC %EV = 84.4%; (**c**) TAG and DAG molecular groups (PC %EV =84.6%; (**d**) FA content in DAG and TAGs (PC %EV = 83.1%).

Principal component analyses (Figure 2) based on TAG species composition dataset—67 variables (Figure 2b) and DAG species composition dataset—13 variables (Figure 2a) yielded the two-component (R^2^X = 0.845 and Q^2^ = 0.654) and the five-component model (R^2^X = 0.996, Q^2^ = 0.967), respectively. The sum of the two first PCs accounted for 97.5% of the variance in DAG content and for 84.4% of variance in TAG content in oils. In the case of DAG subspecies—18 variables—the five-component model (R^2^X = 0.994; Q^2^ = 0.923) was generated, where 97.6% of the variability was explained by the first two components. For the combined DAG and TAG molecular group dataset (80 variables), the PCA gave a two-component model (R^2^X = 0.846; Q^2^ = 0.661) (Figure 2c). The sum of the two first principal components accounted for 84.6% of the total variance. When principal component analysis was performed on the fatty acid profiles of DAG and TAG—396 variables (20 FA in DAGs and 376 FA in TAGs)—a two-component model was obtained with R^2^X = 0.831 and Q^2^ = 0.715 (Figure 2d). The sum of the two first PCs accounted for 83.1% of the total variance.

Samples of the same type of oil (CA, FL and HP) grouped together in different parts of PCA t[1]–t[2] score plots in all models. Clear separation of CA, FL and HP samples was observed indicating high potential of classification and discriminating power of lipidomic profiles.

Next, OPLS-DA models were built on the same datasets. OPLS separates the systematic variation in dataset into two parts, one part that is correlated (predictive) to Y (oil type), and one part that is uncorrelated (orthogonal) to Y. In the single-Y case, there is only one predictive component, and all components beyond the first one reflect orthogonal variation. However, with multiple Y-variables, there can be more than one predictive OPLS component. Each OPLS-DA modeling resulted in the model with two predictive components (2+0+0), which is consistent with the PCA-based observations. Model R^2^X, R^2^Y and Q^2^ were, respectively, equal to 0.844, 0.967 and 0.954 for a TAG species dataset; 0.974, 0.967 and 0.951 for a DAG species dataset; 0.976, 0.965 and 0.952 for a DAG subspecies dataset; 0.845, 0.968 and 0.955 for a combined DAG and TAG species dataset; and 0.831, 0.976 and 0.964 for a FA in DAG and TAG dataset. Clear classification and discrimination of the three examined oils (CA, FL and HP) were observed in the score plots. Models indicated discriminating variables (lipidomic markers). Biplots of OPLS-DA models, which display superimposed scores and loadings, are presented in Figure S1 (Supplementary Materials).

## 3. Discussion

Our results demonstrate that rapid and straightforward analysis by mass spectrometry shotgun lipidomics, followed by data processing using chemometric tools, can quickly classify and distinguish CA, FL, and HP oils according to their specific lipid profiles. Shotgun lipidomics is a high-throughput approach enabling the qualitative and quantitative detection of lipid molecular species in a single run, although this method cannot separate compounds to the extent achieved by coupled techniques, e.g., LC/MS or GC/MS. Moreover, it is incapable to determine regioisomers, or enantiomers, neither to provide structural information on double bond and *sn* positions for TAGs and DAGs.

Initial analysis of group profiles for DAG and TAG contents based on the number of double bonds in FAs and the lengths of FA chains indicated that they were significantly different for the three oils, suggesting the possibility of differentiation of oils (Figure 1). However, more reliable and unequivocal differentiation was achieved by chemometric analysis of lipid profiles (DAG and TAG).

HP, CA and FL oils were clearly differentiated using multivariate analysis. Samples of the same type of oil clearly grouped together in different parts of the PCA space, regardless of the depth level of TAG and DAG characterization. It was found that the oils discrimination can be reached at both the DAG and TAG molecular groups (species), as well as at DAG individual molecule (subspecies) and FA profile in DAG and TAG levels. The two first PCs of the models explained between 83% to 95% of variability in lipids profiles, which can be attributed in each case to between type-of-oil variation. This was confirmed in OPLS-DA analyses upon supervised data modeling, with oil samples assigned to the class (HP, CA and FL), was performed; OPLS-DA models had two predictive components. Unambiguous classification and discrimination of the oils was obtained (Figure S1). The findings from our discovery studies indicated that the profiling (qualitative or quantitative) of these oils for quality control, differentiation, or authenticity may be based on their DAG or TAG, and they will be equally successful. The validation studies involving higher numbers of samples should follow to increase confidence and set the acceptance criteria. The characterization of DAGs and TAGs to species level seems to be sufficient; their more-in-depth characterization to subspecies level or FA profiling in TAGs seems to be not crucial for oil authenticity control and the detection of adulteration. Thus, the fragmentation of the lipid molecules, which delivers subspecies information, i.e., the acyl chain (fatty acid) composition of the lipid molecule, is not imperative, although it is recommended when high analysis confidence is needed. MS mode, providing only species information, i.e., the sum of the carbon atoms and double bonds in the hydrocarbon moieties, may fit the purpose for many applications.

The obtained results indicated that the development of analytical strategies for profiling these three oils using a separation technique (liquid chromatography, LC) may focus only on the DAG class or only on the major components of oils—TAGs—which will grant shorter analysis times and higher laboratory throughput than the oil authenticity control based on comprehensive LC lipid profiling. The acquiring of highly comprehensive lipidomic profiles covering DAG and TAG require high separation selectivity and long chromatographic run times. Currently our undergoing investigations are focused on translating the findings to user-friendly LC-MS based methods.

**Figure 3:**
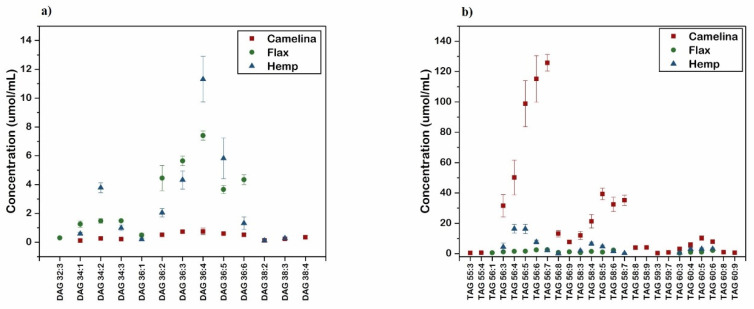
The DAG (**a**) and TAG (**b**) possible differentiation markers of the three oils.

The DAG and TAG molecular groups with the most significant contributions to the differentiation of the three oils are presented in Figure 3. Identified lipid features could be used as qualitative or quantitative markers for differentiating CA, HP and FL oils. For example, CA oil reveals its specific regions of DAGs (36:X) and TAGs (56:X, 58:X and 60:X), which can be used as characteristic markers for CA oil identification. DAGs 34:2; 36:2–6 and TAGs 56:4–5; 58:4 can be considered as quantitative markers for differentiation of all three tested oils, as their concentrations differ significantly between the oils. HP has a very high level of DAG 34:2, DAG 36:4 and DAG 36:5, whereas FL has a high amount of DAG 36:3 and DAG 36:6. CA has a much higher content of TAG 56:3–9 and TAG 58:3–7. On the other hand, specific markers for single oils have been revealed, such as: DAG 32:3 for FL oil, DAG 38:4 for CA oil, TAG 55:3–4 for CA oil, and TAG 56:1 for FL oil. Targeted methods based on the identified lipidomic markers can be developed for the testing of the oils. Various mass spectrometry technologies, both shotgun approaches and coupled to liquid chromatography determinations, may be employed for targeting oil differentiation or specific lipid markers. Cross-instrument adjustment of collision energies is possible [44]. Depending on food testing laboratory needs and their instrument availability, the methods may be based on the markers from the DAG class only, on the markers from the TAG class, or on the markers form both DAG and TAG classes. During method development the precautions should be taken on account of higher sensitivity of coupled techniques, as a lipid not detected under shotgun condition may appear as low intensity peak in a LC/MS chromatogram.

## 4. Conclusions

The study focused on the differentiation of three niche cold-pressed oils (CA, FL, and HP) using a mass spectrometry shotgun lipidomic technique. It provided quantitative DAG and TAG profiles and their FA profiles. It was demonstrated that the identification of each oil sample at the DAG or TAG molecular group level, the DAG subspecies level, and at the FA composition in DAG and TAG level was possible. In each case, multivariate data analyses lead to univocal classification and differentiation of the oils. Differentiating lipid markers were identified and may be used for the development of targeted methods for oil testing. DAGs 34:2; 36:2–6 and TAGs 56:4–5; 58:4 can be employed as quantitative markers for differentiation of all three tested oils, whereas DAG 32:3 for FL oil, DAG 38:4 for CA oil, TAG 55:3–4 for CA oil, and TAG 56:1 for FL oil may be used as oil specific markers.

Targeted approaches including multiple reaction monitoring on liquid chromatography triple quadrupole mass spectrometry systems or a list of targets for easy and fast data interpretation for mass spectrometry shotgun analyses, as more user-friendly techniques, are more feasible for implementation to routine laboratory practice. The main advantage of shotgun technique is the lack of chromatographic separation, which makes the shotgun methods fast, cost-effective, and eco-friendly, so their raising popularity and utility is anticipated. The characterization of oils for the quality control using a shotgun lipidomic analysis is a powerful analytical tool applicable to oil authentication, and the complexity of data processing may be easily overcome with the setting of lists of targets.

## Figures and Tables

**Table 1:** Number of molecular lipid groups and individual lipid molecules.

	Lipid Class	Number of Groups/Individuals			
		Camelina	Flax	Hemp	Total
Molecular groups (species)	DAG	11	10	11	13
	TAG	65	52	48	67
Individual molecules (subspecies)	DAG	12	13	15	18

## Data Availability

Data are contained within the article and the Supplementary Materials.

## Supplementary Materials

The following supporting information can be downloaded at https://www.mdpi.com/article/10.3390/molecules27061848/s1, Figure S1: Biplots of OPLS-DA models for DAG molecular group-, DAG subspecies-, TAG molecular group-, combined DAG and TAG molecular group- and FA composition in DAG and TAG datasets; List S1: List of the lipid class-specific internal standards; Table S1: Content of DAG and TAG molecular groups (species) in camelina, flax and hemp oils; Table S2: Content of DAG individual molecules (subspecies) in camelina, flax and hemp oils; Table S3: Fatty acid composition of DAG and TAG molecular groups detected in camelina, flax and hemp seed oils.

## Sample Availability

Samples of the compounds are not available from the authors.
