# Supplementary material for: Shotgun Lipidomic Analysis for Differentiation of Niche Cold Pressed Oils
Source: Molecules. 2022 Mar 12;27(6):1848. doi: 10.3390/molecules27061848 (PMC8949066; doi:10.3390/molecules27061848)
Supplement: Supplementary file 1 [file molecules-27-01848-s001.zip › molecules-1610286-supplementary.pdf]

SUPPLEMENTARY DATA

to

Shotgun lipidomic analysis for differentiation of niche cold pressed oils

H. Nikolaichuk[1], K. Przykaza[1]*, Anna Kozub[1], Magdalena Montowska[2], Grażyna Wójcicka[3], Jolanta Tomaszewska-Gras[4], E. Fornal[1]

[1] Department of Bioanalytics, Faculty of Biomedicine, Medical University of Lublin, Jaczewskiego 8b, 20-090 Lublin, Poland,

[2] Department of Meat Technology, Poznan University of Life Sciences, ul. Wojska Polskiego 31, 60-624 Poznan, Poland

[3] Department of Pathophysiology, Faculty of Medicine, Medical University of Lublin, Jaczewskiego 8b, 20-090 Lublin, Poland

[4] Department of Food Safety and Quality Management, Faculty of Food Science and Nutrition, Poznan University of Life Sciences, Wojska Polskiego 31/33, 60-624 Poznań, Poland

*Corresponding author address:

Kacper Przykaza
Department of Bioanalytics, Medical University of Lublin, Jaczewskiego 8b, 20-090 Lublin, Poland, e-mail: kacper.przykaza@umlub.pl

Table S1. Content of DAG and TAG molecular groups (spiecies) in camelina, flax and hemp oils.

DAG molecular groups	Camelina		Flax		Hemp	
	Average content [µmol/mL]	Standard deviation [µmol/mL]	Average content [µmol/mL]	Standard deviation [µmol/mL]	Average content [µmol/mL]	Standard deviation [µmol/mL]
32:3	n.d	n.d	0.30	0.03	n.d	n.d
34:1	0.11	0.13	1.27	0.21	0.59	0.11
34:2	0.26	0.06	1.48	0.16	3.78	0.35
34:3	0.21	0.06	1.49	0.07	0.99	0.19
36:1	n.d	n.d	0.49	0.15	0.21	0.06
36:2	0.51	0.06	4.45	0.88	2.05	0.29
36:3	0.73	0.10	5.64	0.33	4.32	0.63
36:4	0.75	0.23	7.41	0.33	11.31	1.59
36:5	0.60	0.07	3.66	0.28	5.82	1.41
36:6	0.51	0.09	4.34	0.34	1.32	0.44
38:2	0.12	0.09	n.d	n.d	0.11	0.11
38:3	0.23	0.05	n.d	n.d	0.28	0.08
38:4	0.34	0.10	n.d	n.d	n.d	n.d

TAG molecular groups	Camelina		Flax		Hemp	
	Average content [µmol/mL]	Standard deviation [µmol/mL]	Average content [µmol/mL]	Standard deviation [µmol/mL]]	Average content [µmol/mL]	Standard deviation [µmol/mL]
50:1	2.16	0.81	1.78	0.19	n.d	n.d
50:2	4.57	1.92	2.04	0.17	3.89	0.55
50:3	5.56	1.84	5.21	0.68	2.39	0.46
50:4	1.11	0.33	0.85	0.08	1.45	0.26
50:5	0.74	0.24	0.42	0.10	0.77	0.09
50:6	0.68	0.17	1.05	0.04	0.13	0.19
51:3	0.22	0.16	n.d	n.d	0.14	0.20
51:4	0.40	0.15	0.39	0.06	0.51	0.31
51:5	0.29	0.21	0.21	0.20	0.44	0.06
51:6	0.32	0.09	0.64	0.10	0.15	0.22
52:2	15.08	5.19	13.49	1.64	n.d	n.d
52:3	24.96	7.70	17.02	1.72	11.19	25.02
52:4	48.14	13.03	58.52	5.47	174.87	33.32
52:5	41.15	11.61	40.81	22.97	114.68	19.36
52:6	38.33	4.84	117.65	9.75	33.64	8.66
52:7	0.51	0.60	1.99	0.20	1.48	1.38
53:3	0.28	0.21	n.d	n.d	n.d	n.d
53:4	0.70	0.18	0.15	0.33	0.79	0.73
53:5	0.63	0.19	0.44	0.40	1.27	0.33
53:6	0.46	0.20	1.43	0.35	0.91	0.20
53:7	n.d	n.d	0.72	0.15	0.07	0.15
54:2	13.11	3.87	3.57	5.17	3.77	1.13
54:3	36.70	10.42	6.69	14.96	n.d	n.d
54:4	61.04	14.75	55.53	7.81	35.95	49.39
54:5	76.71	19.72	57.90	80.42	n.d	n.d
54:6	89.06	21.19	46.88	104.83	420.11	78.09
54:7	100.23	15.79	327.62	20.11	332.56	57.34
54:8	55.05	11.24	234.64	136.78	135.92	21.86
54:9	47.92	3.82	422.44	59.56	42.46	30.35
55:3	0.40	0.11	n.d	n.d	n.d	n.d

55:4	0.65	0.16	n.d	n.d	n.d	n.d
55:5	0.61	0.20	0.10	0.23	n.d	n.d
55:6	0.35	0.26	n.d	n.d	n.d	n.d
56:1	n.d	n.d	0.52	0.12	n.d	n.d
56:2	4.44	3.42	0.46	0.08	1.14	0.76
56:3	31.56	7.33	1.16	0.16	4.42	2.63
56:4	50.15	11.39	1.52	0.22	16.43	2.94
56:5	98.87	15.13	1.61	0.22	16.30	3.08
56:6	115.18	15.35	2.47	0.25	7.64	1.23
56:7	125.79	5.49	2.58	0.31	2.15	0.33
56:8	13.19	2.18	0.75	0.22	0.14	0.20
56:9	7.69	0.62	1.16	0.34	n.d	n.d
57:3	0.40	0.11	n.d	n.d	0.08	0.17
57:4	0.61	0.18	0.07	0.16	0.57	0.54
57:5	0.66	0.19	n.d	n.d	0.38	0.52
57:6	0.53	0.15	0.71	0.11	0.16	0.36
58:2	0.56	1.12	0.36	0.07	0.36	0.26
58:3	11.99	2.60	0.65	0.37	1.94	0.51
58:4	21.29	4.46	1.38	0.13	6.48	1.10
58:5	39.33	3.78	0.95	0.12	4.69	0.86
58:6	32.48	4.71	2.27	0.21	1.53	0.24
58:7	35.16	3.44	n.d	n.d	0.26	0.15
58:8	3.96	0.78	n.d	n.d	n.d	n.d
58:9	4.07	0.49	n.d	n.d	n.d	n.d
59:3	0.31	0.10	n.d	n.d	n.d	n.d
59:4	0.49	0.38	0.09	0.19	0.94	0.16
59:5	0.77	0.24	0.19	0.25	0.80	0.47
59:6	0.34	0.39	0.69	0.08	0.18	0.40
59:7	0.79	0.14	n.d	n.d	n.d	n.d
60:3	3.07	0.75	0.30	0.27	0.53	0.52
60:4	5.68	1.59	0.94	0.14	2.96	0.66
60:5	10.23	1.45	0.96	0.54	3.09	0.59
60:6	7.79	1.10	2.14	0.12	3.13	0.51
60:7	7.23	0.11	1.18	1.10	n.d	n.d
60:8	0.91	0.16	n.d	n.d	n.d	n.d
60:9	0.61	0.04	n.d	n.d	n.d	n.d
61:5	0.17	0.20	n.d	n.d	n.d	n.d

n.d – not detected

Table S2. Content of DAG individual molecules (subspiecies) in camelina, flax and hemp oils.

DAG individual molecules	Camelina		Flax		Hemp	
	Average content [µmol/mL]	Standard deviation [µmol/mL]	Average content [µmol/mL]	Standard deviation [µmol/mL]	Average content [µmol/mL]	Standard deviation [µmol/mL]
14:0/18:3	n.d	n.d	0.30	0.03	n.d	n.d
16:0/18:1	0.11	0.13	1.27	0.21	0.59	0.11
16:0/18:2	0.26	0.06	1.48	0.16	3.79	0.35
16:0/18:3	0.21	0.06	1.49	0.07	0.99	0.19
18:0/18:1	n.d	n.d	0.49	0.15	0.21	0.06
18:0/18:2	n.d	n.d	0.68	0.09	1.32	0.17
18:0/18:3	1.03	0.13	7.54	1.60	1.47	0.49
18:1/18:1	0.03	0.05	0.77	0.14	0.41	0.08
18:1/18:2	0.71	0.06	4.87	0.29	3.91	0.58
18:1/18:3	0.65	0.14	4.29	0.47	2.51	0.47
18:1/20:1	0.20	0.41	6.23	0.95	17.60	2.53
18:2/18:2	0.65	0.14	4.29	0.47	2.51	0.47
18:2/18:3	0.60	0.07	3.66	0.28	5.83	1.41
18:2/20:0	1.02	0.19	8.68	0.68	2.65	0.88
18:2/20:1	0.12	0.09	n.d	n.d	n.d	n.d
18:3/18:3	n.d	n.d	n.d	n.d	0.11	0.11
18:3/20:0	0.12	0.09	n.d	n.d	n.d	n.d
18:3/20:1	0.23	0.05	n.d	n.d	0.19	0.03

n.d – not detected

Table S3. Fatty acid composition of DAG and TAG molecular groups detected in camelina, flax and hemp seed oils.

DAG molecular groups	FA	Camelina		Flax		Hemp	
		Average content [μmol/mL]	Standard deviation [μmol/mL]	Average content [μmol/mL]	Standard deviation [μmol/mL]	Average content [μmol/mL]	Standard deviation [μmol/mL]
32:3	14:0	n.d	n.d	0.30	0.03	n.d	n.d
	18:3	n.d	n.d	0.30	0.03	n.d	n.d
34:1	16:0	0.11	0.13	1.27	0.21	0.59	0.11
	18:1	0.11	0.13	1.27	0.21	0.59	0.11
34:2	16:0	0.26	0.06	1.48	0.16	3.79	0.35
	18:2	0.26	0.06	1.48	0.16	3.79	0.35
34:3	16:0	0.21	0.06	1.49	0.07	0.99	0.19
	18:3	0.21	0.06	1.49	0.07	0.99	0.19
36:1	18:0	n.d	n.d	0.49	0.15	0.21	0.06
	18:1	n.d	n.d	0.49	0.15	0.21	0.06
36:2	18:0	n.d	n.d	0.68	0.09	1.32	0.17
	18:1	1.03	0.13	7.54	1.60	1.47	0.49
	18:2	n.d	n.d	0.68	0.09	1.32	0.17
36:3	18:0	0.03	0.05	0.77	0.14	0.41	0.08
	18:1	0.71	0.06	4.87	0.29	3.91	0.58
	18:2	0.71	0.06	4.87	0.29	3.91	0.58
	18:3	0.03	0.05	0.77	0.14	0.41	0.08
36:4	18:1	0.65	0.14	4.29	0.47	2.51	0.47
	18:2	0.20	0.41	6.23	0.95	17.60	2.53
	18:3	0.65	0.14	4.29	0.47	2.51	0.47
36:5	18:2	0.60	0.07	3.66	0.28	5.83	1.41
	18:3	0.60	0.07	3.66	0.28	5.83	1.41
36:6	18:3	1.02	0.19	8.68	0.68	2.65	0.88
38:2	18:2	0.12	0.09	n.d	n.d	n.d	n.d
	18:2	n.d	n.d	n.d	n.d	0.11	0.11
	20:0	n.d	n.d	n.d	n.d	0.11	0.11
	20:1	0.12	0.09	n.d	n.d	n.d	n.d
38:3	18:2	0.23	0.05	n.d	n.d	0.19	0.03
	18:3	n.d	n.d	n.d	n.d	0.09	0.05
	20:0	n.d	n.d	n.d	n.d	0.09	0.05
	20:1	0.23	0.05	n.d	n.d	0.19	0.03
38:4	18:3	0.34	0.10	n.d	n.d	n.d	n.d
	20:1	0.34	0.10	n.d	n.d	n.d	n.d

TAG molecular groups	FA	Camelina		Flax		Hemp	
		Average content [μmol/mL]	Standard deviation [μmol/mL]	Average content [μmol/mL]	Standard deviation [μmol/mL]	Average content [μmol/mL]	Standard deviation [μmol/mL]
50:1	16:0	4.83	1.78	3.93	0.45	n.d	n.d
	18:1	1.61	0.61	1.40	0.14	n.d	n.d
50:2	14:0	0.18	0.13	0.03	0.07	0.02	0.05
	16:0	9.37	3.92	3.41	1.95	8.27	1.14
	16:1	0.28	0.11	1.17	2.40	n.d	n.d
	18:1	0.49	0.19	0.16	0.15	0.06	0.13
	18:2	3.38	1.46	1.20	0.69	3.31	0.51
	14:0	0.35	0.28	0.09	0.13	0.07	0.15

50:3	16:0	10.33	3.17	10.44	1.46	3.55	0.87
	16:1	0.57	0.23	0.15	0.09	0.76	0.14
	18:1	0.36	0.16	0.16	0.03	0.34	0.07
	18:2	0.76	0.33	0.29	0.05	1.18	0.19
	18:3	4.19	1.27	4.42	0.56	1.24	0.36
50:4	14:0	0.65	0.20	0.52	0.06	1.10	0.19
	16:0	0.51	0.14	0.36	0.05	0.50	0.09
	16:1	0.44	0.13	0.31	0.04	0.28	0.05
	16:2	n.d	n.d	n.d	n.d	0.11	0.07
	18:1	0.37	0.10	0.35	0.05	0.07	0.09
	18:2	0.52	0.20	0.19	0.03	1.79	0.31
	18:3	0.76	0.21	0.77	0.10	0.50	0.10
50:5	14:0	0.55	0.40	0.36	0.21	0.69	0.05
	14:1	0.08	0.09	n.d	n.d	0.14	0.13
	18:2	0.53	0.37	0.34	0.04	0.66	0.03
	18:3	0.92	0.26	0.50	0.09	0.74	0.05
50:6	14:0	0.67	0.16	1.12	0.08	0.12	0.18
	18:3	1.27	0.32	1.98	0.09	0.28	0.38
51:3	17:1	0.16	0.18	n.d	n.d	0.04	0.09
	18:1	0.11	0.13	n.d	n.d	0.12	0.18
	18:2	0.13	0.15	n.d	n.d	0.08	0.19
51:4	15:0	0.23	0.17	0.29	0.04	0.11	0.25
	15:1	0.11	0.07	n.d	n.d	0.06	0.08
	16:0	0.11	0.08	0.15	0.03	0.35	0.39
	17:1	0.06	0.07	0.07	0.07	0.03	0.06
	17:2	n.d	n.d	n.d	n.d	0.11	0.10
	18:1	0.18	0.06	0.19	0.04	0.02	0.05
	18:2	0.29	0.09	0.03	0.07	0.58	0.53
	18:3	0.17	0.12	0.33	0.06	0.21	0.27
51:5	15:0	0.22	0.16	0.07	0.15	0.26	0.08
	15:1	0.13	0.15	0.24	0.26	0.24	0.14
	18:2	0.24	0.19	0.24	0.25	0.41	0.13
	18:3	0.26	0.20	0.08	0.18	0.33	0.10
51:6	15:0	0.24	0.10	0.66	0.12	0.11	0.15
	15:1	0.17	0.13	0.03	0.07	0.08	0.18
	18:3	0.51	0.17	1.22	0.23	0.24	0.33
52:2	16:0	18.37	6.51	16.50	2.31	n.d	n.d
	18:1	25.81	8.53	23.46	3.33	n.d	n.d
52:3	14:0	0.15	0.17	n.d	n.d	n.d	n.d
	16:0	27.89	8.63	n.d	n.d	n.d	n.d
	16:1	0.23	0.27	0.57	0.16	0.37	0.84
	18:0	2.09	1.48	11.13	4.33	n.d	n.d
	18:1	22.82	7.30	17.75	10.06	33.19	74.22
	18:2	21.56	7.48	21.60	3.01	n.d	n.d
	20:1	0.15	0.12	n.d	n.d	n.d	n.d
52:4	14:0	0.28	0.07	n.d	n.d	n.d	n.d
	16:0	50.25	13.68	64.29	8.00	207.53	41.45
	16:1	0.62	0.45	0.46	0.43	0.54	0.75
	18:0	0.04	0.08	0.09	0.08	n.d	n.d
	18:1	26.88	5.62	43.27	7.41	8.66	12.15
	18:2	36.20	13.54	13.70	12.82	307.90	65.90
	18:3	29.81	6.71	53.76	7.91	n.d	n.d
	20:1	0.35	0.04	n.d	n.d	n.d	n.d

52:5	16:0	42.46	11.96	42.55	24.01	119.36	20.65
	16:1	0.88	0.66	0.47	0.65	1.45	1.98
	18:1	0.90	0.65	0.44	0.61	0.74	1.02
	18:2	35.43	10.54	32.83	18.57	103.28	18.56
	18:3	43.76	12.47	46.12	25.92	119.20	20.46
52:6	16:0	39.18	4.57	124.00	11.05	32.64	8.66
	16:1	1.31	0.41	0.79	0.73	2.80	0.45
	16:2	0.04	0.08	n.d	n.d	0.48	0.28
	18:2	2.23	0.59	2.33	2.14	6.19	1.06
	18:3	72.17	8.89	225.82	20.05	58.82	16.77
52:7	16:1	0.41	0.51	2.08	0.28	0.75	0.74
	18:3	1.13	1.31	3.89	0.37	3.69	3.43
53:3	17:1	0.10	0.07	n.d	n.d	n.d	n.d
	18:1	0.26	0.18	n.d	n.d	n.d	n.d
	18:2	0.23	0.16	n.d	n.d	n.d	n.d
	19:1	0.13	0.10	n.d	n.d	n.d	n.d
53:4	17:0	0.26	0.19	n.d	n.d	0.56	0.77
	17:1	0.11	0.13	n.d	n.d	n.d	n.d
	18:1	0.24	0.18	0.18	0.39	0.15	0.33
	18:2	0.45	0.18	0.11	0.25	1.53	1.47
	18:3	0.24	0.18	n.d	n.d	n.d	n.d
	19:1	0.22	0.10	n.d	n.d	n.d	n.d
	19:2	0.31	0.14	n.d	n.d	n.d	n.d
	20:1	0.08	0.10	n.d	n.d	n.d	n.d
53:5	15:1	0.05	0.06	n.d	n.d	n.d	n.d
	17:0	0.23	0.08	0.06	0.13	0.63	0.22
	17:1	0.22	0.09	0.04	0.09	0.49	0.18
	17:2	n.d	n.d	0.05	0.06	0.09	0.05
	18:1	0.17	0.12	0.28	0.31	0.21	0.07
	18:2	0.34	0.14	0.40	0.44	1.25	0.44
	18:3	0.43	0.14	0.10	0.22	0.68	0.24
	19:1	n.d	n.d	0.29	0.32	0.03	0.06
	19:2	0.28	0.02	0.09	0.21	0.30	0.20
	19:3	n.d	n.d	n.d	n.d	0.09	0.09
53:6	17:0	0.24	0.07	1.26	0.41	0.16	0.11
	17:1	0.16	0.11	0.06	0.12	0.42	0.11
	17:2	0.07	0.08	0.06	0.08	0.18	0.26
	18:2	0.22	0.17	0.22	0.20	1.08	0.28
	18:3	0.63	0.22	2.66	0.90	0.82	0.19
53:7	17:1	n.d	n.d	0.65	0.09	n.d	n.d
	18:3	n.d	n.d	1.35	0.21	0.08	0.18
54:2	16:0	8.78	2.28	n.d	n.d	n.d	n.d
	18:0	6.58	2.29	4.05	5.86	4.51	1.33
	18:1	16.09	4.94	6.37	9.11	6.81	2.07
	20:1	7.45	1.86	n.d	n.d	n.d	n.d
54:3	16:0	15.94	3.78	n.d	n.d	n.d	n.d
	18:0	10.17	3.35	4.03	9.01	n.d	n.d
	18:1	50.61	14.65	13.83	30.93	n.d	n.d
	18:2	18.60	5.76	2.21	4.94	n.d	n.d
	20:1	13.94	3.90	n.d	n.d	n.d	n.d
	20:2	0.86	0.25	n.d	n.d	n.d	n.d
	16:0	20.07	3.19	0.15	0.16	0.07	0.15
	18:0	16.99	4.15	33.80	6.42	n.d	n.d

54:4	18:1	59.74	17.14	69.17	9.02	48.78	67.04
	18:2	38.42	13.74	30.95	5.77	55.56	76.08
	18:3	26.84	3.72	32.44	6.69	3.40	7.60
	20:1	18.61	2.40	0.08	0.07	0.04	0.10
	20:2	1.85	0.69	n.d	n.d	n.d	n.d
	20:3	0.41	0.17	n.d	n.d	n.d	n.d
54:5	16:0	2.29	1.67	n.d	n.d	n.d	n.d
	16:1	0.78	0.19	n.d	n.d	n.d	n.d
	16:2	0.06	0.06	n.d	n.d	n.d	n.d
	18:0	14.98	3.78	23.70	33.27	n.d	n.d
	18:1	90.31	21.23	n.d	n.d	n.d	n.d
	18:2	64.14	23.34	58.53	80.23	n.d	n.d
	18:3	53.37	10.31	91.46	128.68	n.d	n.d
	20:1	0.62	0.43	n.d	n.d	n.d	n.d
	20:2	2.62	0.63	n.d	n.d	n.d	n.d
	20:3	0.94	0.29	n.d	n.d	n.d	n.d
54:6	16:0	0.82	0.96	n.d	n.d	n.d	n.d
	18:0	13.05	1.16	n.d	n.d	7.78	9.31
	18:1	64.49	14.81	30.36	67.89	149.67	25.71
	18:2	93.74	29.48	39.28	87.83	905.90	184.09
	18:3	93.95	18.20	71.01	158.78	196.99	37.58
	20:3	1.14	1.32	n.d	n.d	n.d	n.d
54:7	18:1	61.74	5.53	224.97	31.19	42.94	14.12
	18:2	76.28	21.92	176.03	40.44	554.98	108.14
	18:3	162.68	21.59	581.86	45.62	399.76	65.28
54:8	18:2	51.05	10.94	209.24	122.74	132.91	21.92
	18:3	114.12	22.80	494.69	287.63	274.86	44.14
54:9	18:3	143.76	11.46	1267.33	178.69	127.39	91.06
55:3	17:1	0.07	0.08	n.d	n.d	n.d	n.d
	18:1	0.35	0.06	n.d	n.d	n.d	n.d
	18:2	0.18	0.12	n.d	n.d	n.d	n.d
	19:1	0.23	0.27	n.d	n.d	n.d	n.d
	20:1	0.19	0.14	n.d	n.d	n.d	n.d
55:4	17:0	0.14	0.02	n.d	n.d	n.d	n.d
	18:2	0.14	0.17	n.d	n.d	n.d	n.d
	18:3	0.25	0.05	n.d	n.d	n.d	n.d
	19:0	0.30	0.21	n.d	n.d	n.d	n.d
	19:1	0.39	0.11	n.d	n.d	n.d	n.d
	19:2	0.34	0.08	n.d	n.d	n.d	n.d
	20:1	0.23	0.05	n.d	n.d	n.d	n.d
55:5	17:1	0.18	0.05	n.d	n.d	n.d	n.d
	18:3	0.42	0.13	n.d	n.d	n.d	n.d
	19:1	0.49	0.39	n.d	n.d	n.d	n.d
	19:2	0.53	0.09	0.29	0.65	n.d	n.d
	20:1	0.17	0.11	n.d	n.d	n.d	n.d
55:6	17:2	0.08	0.09	n.d	n.d	n.d	n.d
	19:2	0.90	0.74	n.d	n.d	n.d	n.d
56:1	16:0	n.d	n.d	0.19	0.23	n.d	n.d
	18:0	n.d	n.d	0.33	0.15	n.d	n.d
	18:1	n.d	n.d	0.41	0.31	n.d	n.d
	20:0	n.d	n.d	0.31	0.23	n.d	n.d
	22:1	n.d	n.d	0.29	0.32	n.d	n.d
	16:0	2.07	1.52	0.06	0.08	0.05	0.07

56:2	18:0	1.90	3.81	0.08	0.08	n.d	n.d
	18:1	6.23	7.27	0.65	0.21	2.22	1.41
	20:0	2.18	2.55	0.46	0.14	1.12	1.10
	22:1	0.92	1.11	n.d	n.d	n.d	n.d
56:3	16:0	2.16	2.50	0.28	0.03	0.02	0.05
	18:0	3.58	4.14	0.32	0.05	0.10	0.22
	18:1	50.45	7.13	0.74	0.14	6.89	4.89
	18:2	3.68	7.37	0.32	0.06	2.40	3.30
	18:3	n.d	n.d	0.52	0.09	n.d	n.d
	20:0	1.93	3.85	0.64	0.10	2.47	3.40
	20:1	31.71	4.41	0.39	0.07	1.31	0.89
	20:2	0.28	0.34	n.d	n.d	n.d	n.d
	22:0	n.d	n.d	0.26	0.03	n.d	n.d
56:4	16:0	4.09	0.47	n.d	n.d	n.d	n.d
	18:0	7.11	0.66	0.24	0.04	0.06	0.10
	18:1	41.37	9.02	0.99	0.15	4.33	0.85
	18:2	40.89	11.56	0.59	0.11	24.16	3.67
	18:3	n.d	n.d	1.01	0.22	1.69	1.59
	20:0	10.89	3.24	1.11	0.22	15.22	2.44
	20:1	43.23	8.70	0.57	0.05	3.63	0.72
	20:2	2.62	0.66	n.d	n.d	0.18	0.03
	20:3	0.09	0.10	n.d	n.d	n.d	n.d
	22:2	0.06	0.07	n.d	n.d	n.d	n.d
	22:3	0.11	0.08	n.d	n.d	n.d	n.d
56:5	16:0	0.38	0.34	n.d	n.d	n.d	n.d
	18:1	52.67	4.19	0.73	0.09	1.15	0.68
	18:2	70.26	18.14	0.84	0.15	19.55	3.90
	18:3	67.67	8.20	1.40	0.20	9.69	1.91
	20:0	12.40	2.72	0.75	0.09	9.06	1.97
	20:1	85.40	11.03	0.98	0.14	9.13	1.82
	20:2	5.74	1.88	0.06	0.05	0.29	0.28
	20:3	1.26	0.91	n.d	n.d	0.01	0.01
	22:3	0.37	0.32	n.d	n.d	n.d	n.d
56:6	16:0	0.32	0.21	n.d	n.d	n.d	n.d
	18:0	0.45	0.42	n.d	n.d	n.d	n.d
	18:1	9.75	1.29	0.16	0.10	0.14	0.13
	18:2	85.78	12.84	0.91	0.17	4.64	2.72
	18:3	128.04	14.68	3.47	0.41	10.41	6.14
	20:0	12.94	0.28	1.54	0.32	2.57	1.63
	20:1	95.10	12.90	0.97	0.17	4.25	2.51
	20:2	10.39	1.99	0.22	0.05	0.88	0.53
	20:3	2.75	1.89	0.12	0.07	0.02	0.05
56:7	18:1	5.08	0.45	0.21	0.12	n.d	n.d
	18:2	14.75	2.79	0.36	0.10	0.50	0.46
	18:3	222.97	10.47	4.30	0.52	3.62	1.19
	20:1	116.96	6.66	2.18	0.28	1.72	0.55
	20:2	10.21	2.09	0.30	0.09	0.41	0.38
	20:3	7.38	0.63	0.38	0.03	0.18	0.13
56:8	18:2	4.69	0.79	0.26	0.07	0.17	0.32
	18:3	20.47	3.38	1.15	0.37	0.13	0.28
	20:2	8.58	1.40	0.50	0.14	0.05	0.12
	20:3	5.79	0.95	0.33	0.09	0.03	0.08
	18:3	14.60	1.26	2.21	0.66	n.d	n.d

56:9	20:3	8.35	0.78	1.26	0.36	n.d	n.d
57:3	19:0	0.24	0.17	n.d	n.d	n.d	n.d
	19:1	0.71	0.38	n.d	n.d	0.23	0.52
	19:2	0.13	0.10	n.d	n.d	n.d	n.d
57:4	19:0	0.47	0.20	0.04	0.09	0.23	0.51
	19:1	0.66	0.25	0.14	0.32	0.39	0.87
	19:2	0.51	0.06	0.03	0.08	0.59	0.81
	21:1	0.15	0.12	n.d	n.d	n.d	n.d
57:5	19:1	1.03	0.41	n.d	n.d	0.40	0.55
	19:2	0.86	0.24	n.d	n.d	0.73	1.05
57:6	18:3	n.d	n.d	0.12	0.07	n.d	n.d
	19:1	0.38	0.34	n.d	n.d	n.d	n.d
	19:2	1.13	0.19	n.d	n.d	0.49	1.08
	21:0	n.d	n.d	1.99	0.35	n.d	n.d
58:2	18:1	n.d	n.d	0.64	0.13	0.59	0.75
	22:0	0.33	0.66	0.45	0.07	0.14	0.20
58:3	18:0	0.38	0.75	0.90	0.54	n.d	n.d
	18:1	12.98	2.68	n.d	n.d	5.03	1.12
	18:2	n.d	n.d	0.71	0.66	n.d	n.d
	20:1	17.20	2.86	n.d	n.d	0.27	0.11
	22:1	4.85	1.15	0.16	0.15	0.45	0.60
58:4	18:0	1.20	0.10	n.d	n.d	n.d	n.d
	18:1	7.83	1.74	0.98	0.13	1.07	0.34
	18:2	15.43	3.81	0.40	0.25	10.67	1.56
	18:3	n.d	n.d	1.21	0.12	0.32	0.72
	20:0	2.40	1.63	n.d	n.d	n.d	n.d
	20:1	25.30	4.84	n.d	n.d	0.07	0.10
	20:2	1.52	1.07	n.d	n.d	n.d	n.d
	22:0	2.07	0.42	1.35	0.11	6.79	1.06
	22:1	7.34	1.77	0.07	0.07	0.36	0.35
	22:2	0.26	0.10	0.11	0.24	0.13	0.29
58:5	18:1	9.42	1.33	n.d	n.d	0.17	0.32
	18:2	17.01	4.20	2.46	0.36	4.43	0.74
	18:3	26.60	1.68	n.d	n.d	4.43	0.70
	20:0	1.29	0.27	n.d	n.d	n.d	n.d
	20:1	38.68	2.40	n.d	n.d	0.06	0.09
	20:2	5.91	1.24	n.d	n.d	n.d	n.d
	20:3	0.64	0.75	n.d	n.d	n.d	n.d
	22:0	2.43	0.35	n.d	n.d	4.06	0.66
	22:1	15.35	2.68	0.36	0.07	0.90	0.35
	22:3	0.49	0.04	n.d	n.d	n.d	n.d
58:6	18:0	0.19	0.03	n.d	n.d	n.d	n.d
	18:1	1.31	0.30	n.d	n.d	n.d	n.d
	18:2	17.74	3.28	0.03	0.06	0.62	0.13
	18:3	31.47	4.32	4.38	0.42	2.35	0.32
	20:0	0.62	0.09	n.d	n.d	n.d	n.d
	20:1	11.21	2.15	n.d	n.d	n.d	n.d
	20:2	9.71	1.96	n.d	n.d	n.d	n.d
	20:3	3.22	0.59	n.d	n.d	n.d	n.d
	22:0	2.52	0.18	2.38	0.25	0.95	0.18
	22:1	18.07	2.95	0.03	0.07	0.63	0.17
	22:2	0.70	0.52	n.d	n.d	n.d	n.d
	22:3	0.69	0.47	n.d	n.d	n.d	n.d

58:7	18:1	1.19	0.06	n.d	n.d	n.d	n.d
	18:2	3.61	0.64	n.d	n.d	n.d	n.d
	18:3	54.68	5.19	n.d	n.d	0.48	0.28
	20:1	7.41	1.48	n.d	n.d	n.d	n.d
	20:2	1.46	0.26	n.d	n.d	n.d	n.d
	20:3	7.88	1.21	n.d	n.d	n.d	n.d
	20:4	0.07	0.09	n.d	n.d	n.d	n.d
	22:1	25.42	2.10	n.d	n.d	0.27	0.18
	22:2	1.36	0.36	n.d	n.d	n.d	n.d
	22:3	2.37	0.26	n.d	n.d	0.04	0.08
58:8	18:2	2.05	0.37	n.d	n.d	n.d	n.d
	18:3	4.70	0.91	n.d	n.d	n.d	n.d
	20:2	0.67	0.17	n.d	n.d	n.d	n.d
	20:3	0.86	0.19	n.d	n.d	n.d	n.d
	22:2	1.01	0.21	n.d	n.d	n.d	n.d
	22:3	2.53	0.50	n.d	n.d	n.d	n.d
58:9	18:3	7.25	0.79	n.d	n.d	n.d	n.d
	20:3	0.65	0.11	n.d	n.d	n.d	n.d
	22:3	4.07	0.51	n.d	n.d	n.d	n.d
	22:5	0.08	0.09	n.d	n.d	n.d	n.d
59:3	18:1	0.23	0.26	n.d	n.d	n.d	n.d
	19:1	0.39	0.23	n.d	n.d	n.d	n.d
	23:1	0.14	0.16	n.d	n.d	n.d	n.d
59:4	18:2	0.11	0.23	0.06	0.14	1.45	0.21
	19:1	0.26	0.28	n.d	n.d	n.d	n.d
	19:2	0.29	0.31	n.d	n.d	n.d	n.d
	21:0	0.48	0.40	n.d	n.d	n.d	n.d
	23:0	0.07	0.15	0.20	0.44	1.20	0.21
59:5	18:2	n.d	n.d	0.36	0.50	0.61	0.37
	18:3	n.d	n.d	n.d	n.d	0.66	0.42
	19:1	0.67	0.78	n.d	n.d	n.d	n.d
	19:2	1.02	0.23	n.d	n.d	n.d	n.d
	21:1	0.57	0.14	n.d	n.d	n.d	n.d
	23:0	n.d	n.d	n.d	n.d	0.81	0.48
	23:1	n.d	n.d	0.19	0.26	0.31	0.19
59:6	18:3	n.d	n.d	1.21	0.14	0.33	0.74
	23:0	n.d	n.d	0.85	0.14	0.21	0.48
59:7	18:3	1.35	0.22	n.d	n.d	n.d	n.d
	23:1	1.01	0.20	n.d	n.d	n.d	n.d
60:3	18:1	3.33	0.91	0.16	0.22	0.92	1.35
	18:2	n.d	n.d	0.29	0.31	n.d	n.d
	20:1	2.48	0.53	n.d	n.d	0.60	1.33
	22:1	2.15	0.55	n.d	n.d	n.d	n.d
	24:1	1.10	0.31	0.02	0.05	0.04	0.10
60:4	18:1	1.94	0.64	0.45	0.41	0.37	0.22
	18:2	4.34	1.27	0.42	0.42	5.07	0.96
	18:3	n.d	n.d	0.52	0.49	n.d	n.d
	20:0	0.20	0.14	n.d	n.d	n.d	n.d
	20:1	3.76	1.07	n.d	n.d	0.06	0.12
	20:2	0.23	0.29	n.d	n.d	n.d	n.d
	22:0	0.32	0.38	n.d	n.d	n.d	n.d
	22:1	3.69	1.00	n.d	n.d	0.07	0.15
	22:2	0.06	0.08	n.d	n.d	n.d	n.d

	24:0	0.97	0.36	1.40	0.46	3.17	0.74
	24:1	1.51	0.52	0.03	0.06	0.17	0.11
60:5	18:1	1.80	0.36	n.d	n.d	n.d	n.d
	18:2	4.26	1.22	2.40	1.36	2.91	0.58
	18:3	7.17	0.84	n.d	n.d	2.98	0.57
	20:1	4.99	0.45	n.d	n.d	0.09	0.21
	20:2	1.44	0.36	n.d	n.d	n.d	n.d
	20:3	0.15	0.17	n.d	n.d	n.d	n.d
	22:0	0.31	0.05	n.d	n.d	n.d	n.d
	22:1	6.01	0.76	n.d	n.d	0.10	0.23
	22:1	0.25	0.04	n.d	n.d	n.d	n.d
	22:3	0.22	0.04	n.d	n.d	n.d	n.d
	24:0	1.06	0.31	n.d	n.d	2.53	0.42
	24:1	2.96	0.78	0.48	0.31	0.66	0.14
60:6	18:1	0.06	0.08	n.d	n.d	n.d	n.d
	18:2	3.60	0.79	0.05	0.11	0.56	0.78
	18:3	7.78	1.12	4.12	0.29	6.00	0.96
	20:1	0.69	0.12	n.d	n.d	n.d	n.d
	20:2	2.34	0.69	n.d	n.d	n.d	n.d
	20:3	0.57	0.43	n.d	n.d	n.d	n.d
	22:0	0.11	0.08	n.d	n.d	n.d	n.d
	22:1	2.90	0.75	n.d	n.d	n.d	n.d
	22:2	0.38	0.11	n.d	n.d	n.d	n.d
	22:3	0.49	0.09	n.d	n.d	n.d	n.d
	24:0	1.01	0.21	2.20	0.24	2.08	1.19
	24:1	3.31	0.87	0.05	0.11	0.67	0.92
	24:2	0.07	0.08	n.d	n.d	0.08	0.17
60:7	18:2	0.48	0.06	n.d	n.d	n.d	n.d
	18:3	10.50	0.61	2.26	2.14	n.d	n.d
	20:1	0.76	0.08	n.d	n.d	n.d	n.d
	20:2	0.35	0.11	n.d	n.d	n.d	n.d
	20:3	1.95	0.37	n.d	n.d	n.d	n.d
	22:1	1.77	0.35	n.d	n.d	n.d	n.d
	22:2	0.20	0.04	n.d	n.d	n.d	n.d
	22:3	0.99	0.11	n.d	n.d	n.d	n.d
	24:1	4.43	0.51	1.29	1.18	n.d	n.d
	24:2	0.10	0.07	n.d	n.d	n.d	n.d
	24:3	0.10	0.07	n.d	n.d	n.d	n.d
60:8	18:2	0.11	0.13	n.d	n.d	n.d	n.d
	18:3	0.79	0.55	n.d	n.d	n.d	n.d
	20:2	0.81	0.41	n.d	n.d	n.d	n.d
	20:3	0.27	0.24	n.d	n.d	n.d	n.d
	22:2	0.06	0.06	n.d	n.d	n.d	n.d
	22:3	0.58	0.41	n.d	n.d	n.d	n.d
60:9	18:3	1.08	0.29	n.d	n.d	n.d	n.d
	20:3	0.24	0.29	n.d	n.d	n.d	n.d
	22:3	0.21	0.25	n.d	n.d	n.d	n.d
	24:3	0.30	0.21	n.d	n.d	n.d	n.d
61:5	19:2	0.11	0.15	n.d	n.d	n.d	n.d
	23:1	0.41	0.49	n.d	n.d	n.d	n.d

n.d – not detected

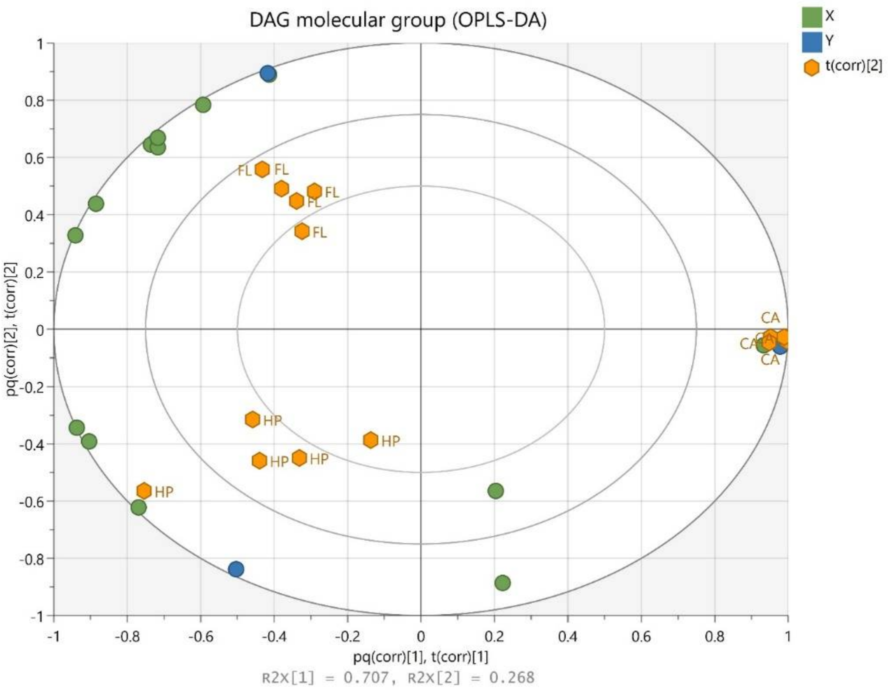
DAG molecular group (OPLS-DA)
X
Y
t(corr)[2]
pq(corr)[2], t(corr)[2]
pq(corr)[1], t(corr)[1]
R2X[1] = 0.707, R2X[2] = 0.268
FL
HP
CA

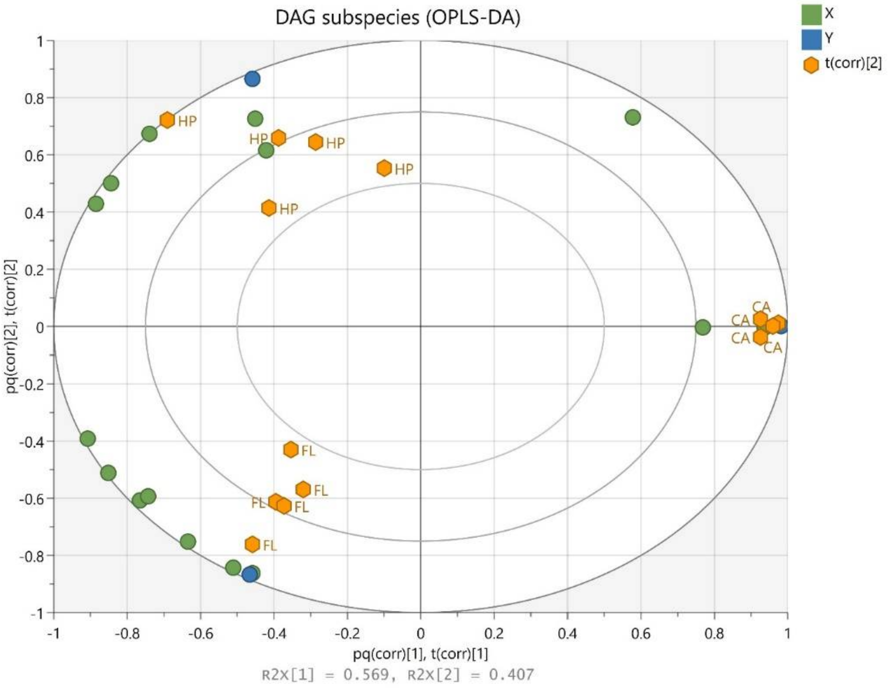
DAG subspecies (OPLS-DA)
X
Y
t(corr)[2]
pq(corr)[2], t(corr)[2]
pq(corr)[1], t(corr)[1]
R2X[1] = 0.569, R2X[2] = 0.407
HP
FL
CA

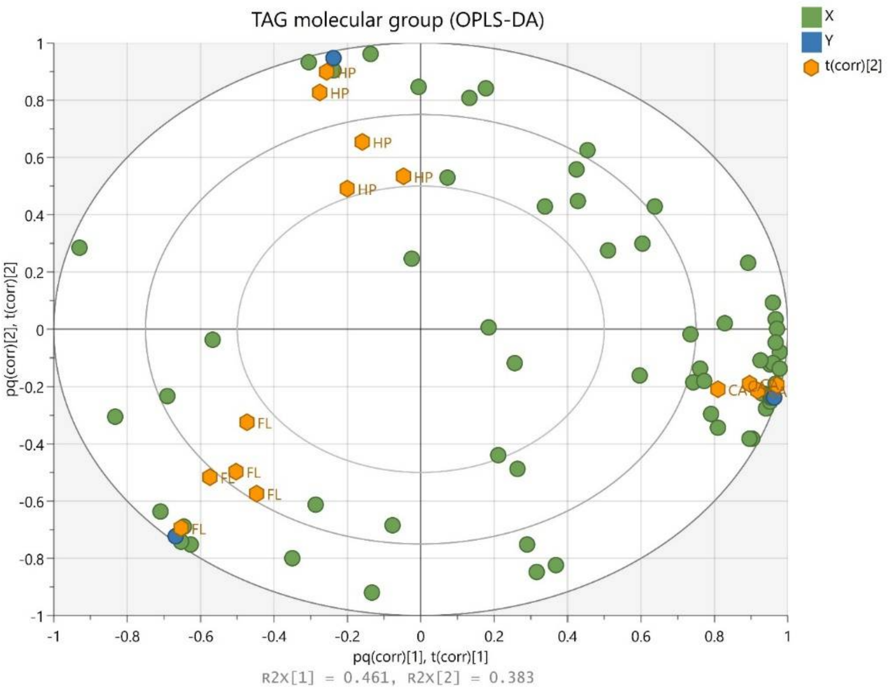
TAG molecular group (OPLS-DA)
X
Y
t(corr)[2]
pq(corr)[2], t(corr)[2]
pq(corr)[1], t(corr)[1]
R2X[1] = 0.461, R2X[2] = 0.383
HP
FL
CA

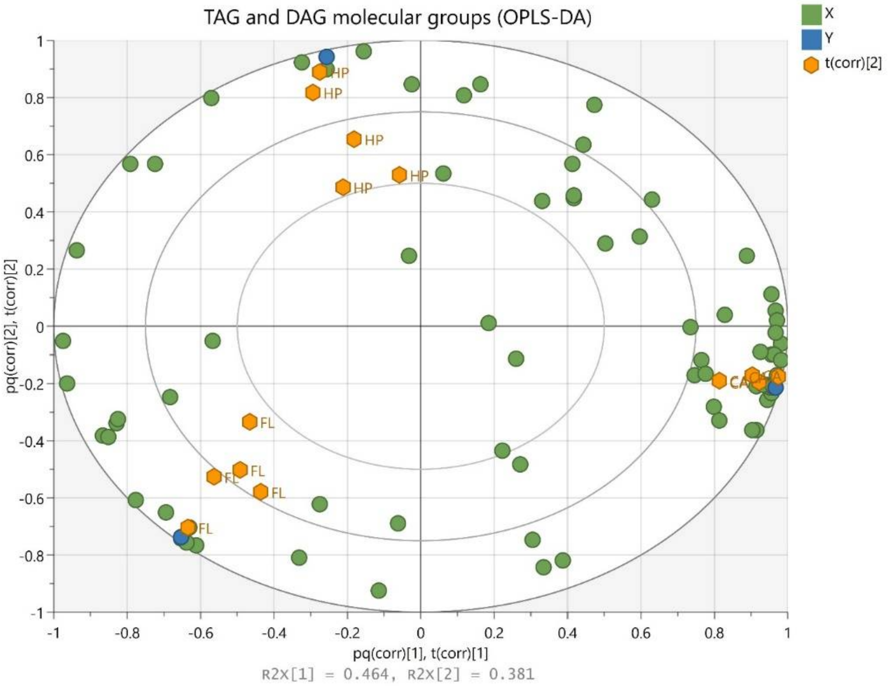
TAG and DAG molecular groups (OPLS-DA)
X
Y
t(corr)[2]
pq(corr)[2], t(corr)[2]
pq(corr)[1], t(corr)[1]
R2X[1] = 0.464, R2X[2] = 0.381
HP
FL
CA

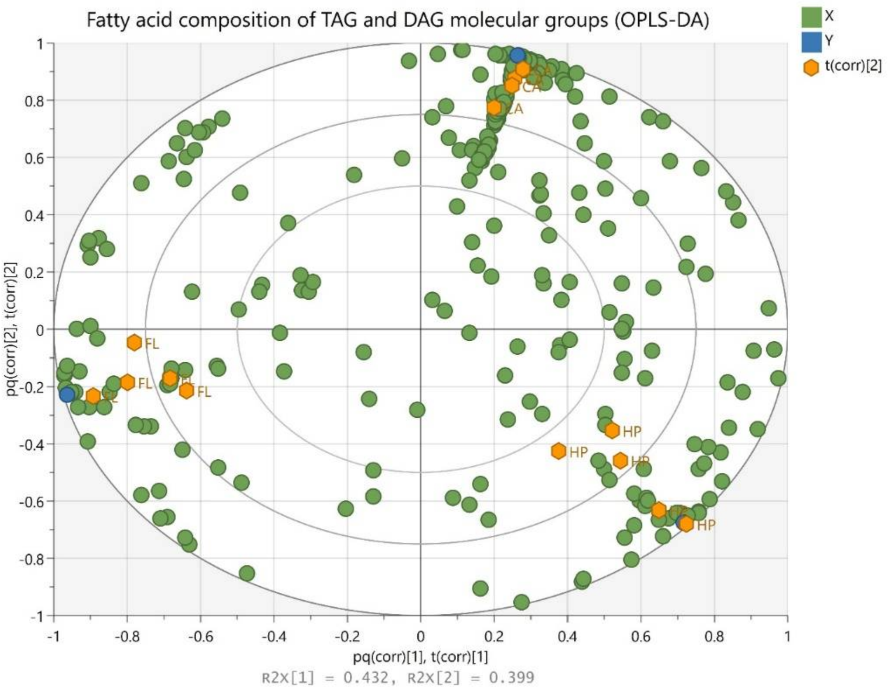

Figure S1. Biplots of OPLS-DA models for DAG molecular grup-, DAG subspiecies-, TAG molecular group-, combined DAG and TAG molecular group- and FA composition in DAG and TAG datasets.

List S1. List of the lipid class-specific internal standards.

50 pmol of lysophasphatidylglycerol (LPG) 17:1, 50 pmol of lysophosphatic acid (LPA) 17:0, 500 pmol of phosphatidylcholine (PC) 17:0/17:0, 30 pmol of hexosylceramide (HexCer) 18:1;2/12:0, 50 pmol of phosphatidylserine (PS) 17:0/17:0, 50 pmol of phosphatidylglycerol (PG) 17:0/17:0, 50 pmol of phosphatic acid (PA) 17:0/17:0, 50 pmol of lysophposphatidylinositol (LPI 17:1), 50 pmol of lysophosphatidylserine (LPS) 17:1, 1 nmol cholesterol (Chol) D6, 100 pmol of diacylglycerol (DAG) 17:0/17:0, 50 pmol of triacylglycerol (TAG) 17:0/17:0/17:0, 50 pmol of ceramide (Cer) 18:1;2/17:0, 200 pmol of sphingomyelin (SM) 18:1;2/12:0, 50 pmol of lysophosphatidylcholine (LPC) 12:0, 30 pmol of lysophosphatidylethanolamine (LPE) 17:1, 50 pmol of phosphatidylethanolamine (PE) 17:0/17:0, 100 pmol of cholesterol ester (CE) 20:0, 50 pmol of phosphatidylinositol (PI) 16:0/16:0
